# High energetic excitons in carbon nanotubes directly probe charge-carriers

**DOI:** 10.1038/srep09681

**Published:** 2015-05-11

**Authors:** Giancarlo Soavi, Francesco Scotognella, Daniele Viola, Timo Hefner, Tobias Hertel, Giulio Cerullo, Guglielmo Lanzani

**Affiliations:** 1 Dipartimento di Fisica, Politecnico di Milano, Piazza L. da Vinci 32, 20133 Milano, Italy; 2 IFN-CNR, Piazza L. da Vinci, 32, 20133 Milano, Italy; 3 Center for Nano Science and Technology@PoliMi, Istituto Italiano di Tecnologia, Via Giovanni Pascoli, 70/3, 20133 Milano, Italy; 4 Inst. for Physical and Theoretical Chemistry Dept. of Chemistry and Pharmacy, University of Wuerzburg, Wuerzburg 97074, Germany

## Abstract

Theory predicts peculiar features for excited-state dynamics in one dimension (1D) that are difficult to be observed experimentally. Single-walled carbon nanotubes (SWNTs) are an excellent approximation to 1D quantum confinement, due to their very high aspect ratio and low density of defects. Here we use ultrafast optical spectroscopy to probe photogenerated charge-carriers in (6,5) semiconducting SWNTs. We identify the transient energy shift of the highly polarizable S_33_ transition as a sensitive fingerprint of charge-carriers in SWNTs. By measuring the coherent phonon amplitude profile we obtain a precise estimate of the Stark-shift and discuss the binding energy of the S_33_ excitonic transition. From this, we infer that charge-carriers are formed instantaneously (<50 fs) even upon pumping the first exciton, S_11_. The decay of the photogenerated charge-carrier population is well described by a model for geminate recombination in 1D.

The study of photo-excitation dynamics in one dimension has been prompted by theoretical predictions of a wealth of singular properties, such as the giant oscillator strength and non-linear response of confined states, the large Coulomb interaction, the sharply-peaked density of states and peculiar excited-state recombination kinetics^1^,^2^,^3^. In this respect, SWNTs represent a very close approximation to a 1D solid, easily achieving aspect ratio as high as 10^3^. Theory predicts that Wannier-Mott excitons are the elementary photoexcitations in SWNTs, due to the strong Coulomb interaction caused by limited screening^4^,^5^. These excitons have typical 1D characteristics: negligible free carrier generation, large binding energy, non-negligible size and 1D transport. Theoretical predictions are supported by several experimental results, such as the measured binding energy, typically 0.1–1 eV^6^,^7^, and the electron-hole correlation length, in the 2–5 nm range^8^. The exciton model alone, however, fails to capture the whole dynamics following photoexcitation, and many other photoexcited species have crowded the complex scenario of SWNTs' optical response, ranging from triplets^9^ to bi-excitons^10^ and trions^11^. Photocurrent^12^,^13^,^14^,^15^,^16^, transient absorption^14^,^17^,^18^ and THz spectroscopy^19^,^20^ experiments also point out a non-negligible photogeneration of free charge-carriers in SWNTs. This is in stark contrast with the excitonic model and the reduced Sommerfeld factor that implies excitons be the only species generated upon photoexcitation. Attempts to solve this discrepancy proposed possible non-linear phenomena^21^ as the mechanism of charge-carrier photogeneration in SWNTs. However, there is solid experimental evidence that the charge-carrier yield is linear with the pump fluence^19^. Besides this, the nature of high energetic transitions in small-diameter semiconducting SWNTs is still matter of debate, given that both excitonic^22^,^23^ and band-to-band transitions^24^,^25^ have been invoked to explain recent experimental results. Here we apply ultrafast optical spectroscopy to the semiconducting (6,5) SWNTs and show that charge-carriers can be identified by their effect on excitonic resonances, in particular the large energy shift that they induce on the third excitonic subband (S_33_) transition. The availability of a good fingerprint for charge-carriers enables us to study their dynamics in one dimension. We find that, upon excitation of the lowest optical transition, a fraction of the absorbed photons generates geminate charge-carrier pairs “instantaneously” (<50 fs). The carriers recombine on the sub-nanosecond timescale following the characteristic kinetic law (~t^−1/2^) of a random walk in 1D. This kinetics is consistent with an initial electron-hole separation of the same order as the exciton correlation length.

## Results

**Figure 1:**
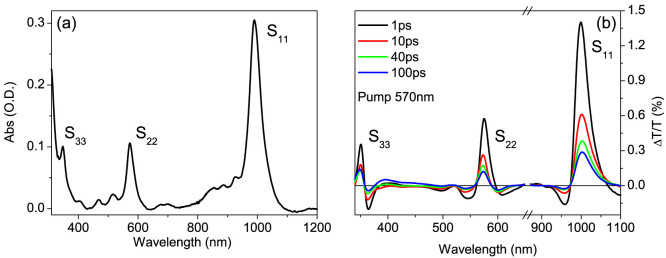
Linear and transient absorption spectra of SWNTs. (a) Absorption spectrum of the enriched (6,5) SWNTs sample. (b) Transient absorption spectra at different pump-probe delays, with 570 nm excitation wavelength, for a (6,5) enriched SWNT sample. The probe is obtained with white light continuum, from CaF_2_ for the wavelengths from 340 nm to 650 nm and from sapphire for wavelengths from 850 nm to 1100 nm.

Figure 1a shows the linear absorption spectrum of the sample with its first three excitonic transitions: S_11_ near 1 μm, S_22_ near 570 nm and S_33_ near 350 nm. Figure 1b shows Δ*T*/*T* spectra for 570 nm excitation wavelength at different pump-probe delays. In agreement with our previous work^14^, we find that the shape of the transient spectral response does not depend on the excitation wavelength (Supplementary material). We observe three sharp positive Δ*T*/*T* peaks corresponding to the three excitonic transitions, each associated with negative features, smaller than the positive one for the first and second excitons, but comparable for the third exciton. The large positive peak in the first exciton region can be assigned to photobleaching (PB) due to state filling. The photoinduced absorption (PA) above 1.1 μm has been tentatively assigned to triplets^26^, metallic tubes^27^, trions^21^, transitions from S_11_ to the first band edge^28^ or bi-excitons^29^. The complex shape of the Δ*T*/*T* signal around S_22_ can be reproduced with a red shift or a broadening of the ground state absorption spectrum. Several processes, such as photoinduced dephasing^30^, bi-exciton formation^31^,^32^, phonon dynamics^33^ and charge induced Stark effect^14^,^18^ have been invoked to explain the transient signal in this spectral region. Both the first and second excitons thus present complex transients, due to the superposition of several overlapping contributions. On the other hand, the third excitonic sub-band shows a simple first derivative lineshape that corresponds to a photoinduced red shift of the ground state transition.

**Figure 2:**
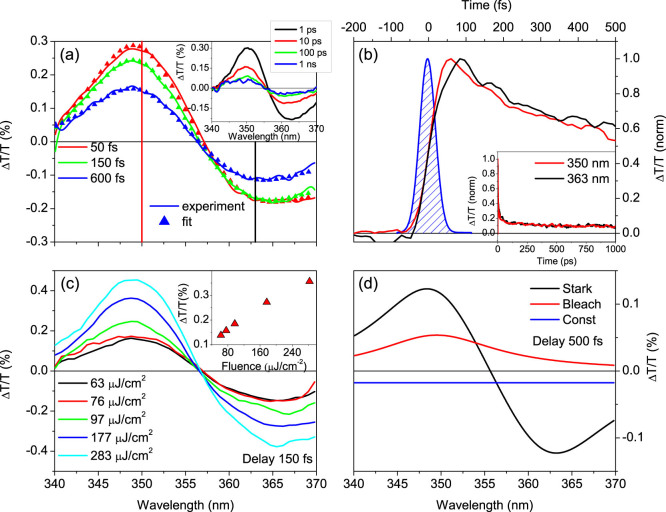
(a) Δ*T*/*T* spectra at different pump-probe delays (solid line) and Δ*T*/*T* spectra obtained from the fitting (triangles). The excitation fluence is approximately 100 μJ/cm^2^ for the main figure and 200 μJ/cm^2^ for the inset. (b) Dynamics for wavelengths on the positive and negative peaks of the signal compared to our temporal resolution (≈50 fs). The pump pulse excites the first excitonic subband S_11_ in the IR region (≈1 μm). (c) Transient spectra at different pump intensities for 150-fs pump-probe delay and (inset) absolute value of the Δ*T*/*T* signal at 363 nm as a function of the pump fluence. (d) Spectral components used for the fitting, i.e. PB, PA and Stark shift, at 500 fs pump-probe delay.

**Figure 3:**
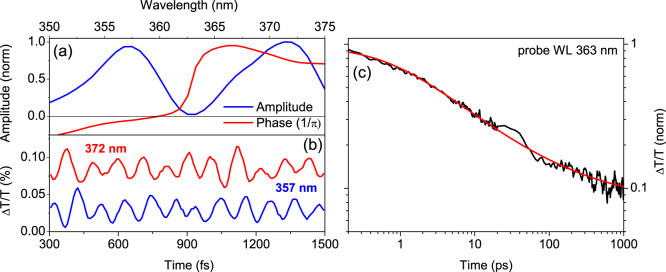
(a) Fourier transform's amplitude and phase for the (6,5) RBMs' frequency (318 cm^−1^) near the S_33_ energy region. (b) Oscillatory component at 357 nm (blue) and 372 nm (red), the two maxima of RBMs oscillations' amplitude. (c) Normalized dynamics at 363 nm (black) and fit (red) with a 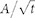 function, where *t* is time and *A* is a fitting parameter. The excitation fluence is approximately 90 μJ/cm^2^.

Figure 2 zooms in on the transient Δ*T*/*T* spectra and dynamics in the region near S_33_ when the sample is excited at the S_11_ transition, with ≈50 fs temporal resolution (Fig. 2b). After rapid initial changes in the first ≈150 fs (Fig. 2a), the shape of the transient spectra remains unvaried up to 1 ns, the longest delay investigated here (inset of Fig. 2a). The observed derivative shape is insensitive to the pump-photon energy (Supplementary material), thus excluding bi-excitons and trions. Intensity dependent measurements (Fig. 2c and Supplementary information) demonstrate that the experiments, at least at lower fluences, are performed in a linear regime, thus ruling out non-linear processes such as two-photon absorption or exciton-exciton annihilation^21^, which is expected to occur in a saturation regime for exciton photogeneration^34^,^35^,^36^. Similarly, the signal is weakly sensitive to changes in temperature (Supplementary material), excluding geometrical re-arrangement (i.e. diameter distortion) and thermal effects as a possible origin of the strong red-shift of S_33_ upon photoexcitation. In order to better understand the origin of the Δ*T*/*T* signal for S_33_, we fit it by the sum of three contributions (Fig. 2d and Supplementary material): a Lorentzian function, corresponding to the ground-state PB, the difference between two Lorentzian functions, corresponding to a spectral red-shift by 0.13 eV, and a constant PA over the entire probe bandwidth. The ΔT/T signal for the third exciton is dominated by the spectral shift that we assign to the Stark shift induced on the S_33_ transition by the intense local electric field of photogenerated charge-carriers. The fit also indicates that PB, PA and Stark shift are all formed within our temporal resolution, as confirmed by the ultrafast build-up of the ΔT/T signal (Fig. 2b) and in good agreement with the claim of instantaneous charge photogeneration of Ref. 20. The PB signal decays faster (≈600 fs) with respect to the Stark signal, as expected for the lifetime of excitons with respect to free or trapped charge-carriers. The fast decay of the PB signal also suggests that at longer delays (i.e. few picoseconds to nanoseconds) the transient signal at the S_33_ transition directly probes charge-carriers. To obtain another precise estimate of the Stark shift of the S_33_ exciton we study the clear periodic temporal modulations of the ΔT/T signal around the S_33_ transition (Fig. 3a), that we assign to impulsively excited coherent phonons, namely the radial breathing modes (RBMs). Their coupling to the optical response of SWNTs can be easily understood: exciton binding energies are approximately inversely proportional to the tube diameter^37^,^38^, so that the exciton absorption peak undergoes red or blue shift according to diameter variations. The resulting oscillations have zero amplitude at the peak of the resonance and maximum amplitude with opposite phase for higher and lower energies^39^. Figure 3b shows the oscillatory component of the Δ*T*/*T* signal for two wavelengths near the S_33_ peak. Fourier transform of the time traces indicates a dominant frequency of 318 cm^−1^ (Supplementary information), consistent with the RBM of the (6,5) SWNT. Figure 3a shows the corresponding amplitude and phase of the modulation versus probe energy. Interestingly, the peak resonance energy, indicated by the zero amplitude and by the phase jump (Fig. 3a), is red-shifted from the ground state S_33_ transition (at 349 nm). In other words, coherent RBM phonons modulate the Stark shifted transition, thus providing a very sensitive tool for measuring the Stark shift of the exciton resonance. We obtain Δ*E_Stark_* ~ 130 *meV*, in excellent agreement with the results of the fitting model.

## Discussion

The Stark effect depends on the transition's polarizability and is enhanced for excitons with small binding energy. In contrast to state filling which selectively affects only transitions involving populated states, the Stark effect lacks this selectivity and affects all optical transitions. Our assignment of a red-shift induced by Stark effect is based on the following chain of reasoning: i) charge-carriers are photogenerated in SWNTs; ii) each charge-carrier is a source of a strong local electric field; iii) the amplitude of the Stark signal has a distinct kinetics from that of the exciton PB, being in particular much longer lived; iv) any other source of modulation that could explain the first derivative shape of the ΔT/T spectra for S_33_ has been ruled out. We propose that photoexcitation at the S_11_ transition creates both excitons and free charge-carriers: the first bleaches S_33_ (due ground state depletion and/or phase space filling), the latter shifts the energy levels due to Stark effect. The Stark effect prevails, making the S_33_ transition privileged to probe charge-carriers, for at least two reasons: i) the S_33_ exciton has small cross-section and thus small PB signal; ii) it has low binding energy^24^,^25^, resulting in large field-induced energy shifts. The latter point deserves detailed attention. In fact, the nature of S_33_ in semiconducting SWNTs is still matter of debate: Rayleigh scattering experiments have demonstrated that it is consistent with an excitonic model^22^, theoretical studies predict high binding energies^23^ while recent experiments show features ascribable to unbound electron-hole pairs^24^,^25^. The PB contribution to the S_33_ transient signal (Fig. 2d) suggests that this transition is excitonic in nature and thus the quadratic part of the Stark effect can be expressed as 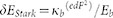, where *κ_b_* is a fitting constant, *e* is the electron charge, *d* the SWNT diameter, *F* the electric field responsible for the Stark shift and *E_b_* the exciton binding energy^40^. Since the Stark effect is inversely proportional to the exciton binding energy, we conclude that the large red shift (≈130 meV) that we observe on the S_33_ exciton is a consequence of its low binding energy (in agreement with Ref. 24), in particular if compared to lower-energy excitons (S_11_ and S_22_), where the Stark shift is considerably smaller. In fact, the observation of a first derivative shape only for the highly polarizable S_33_ transition is readily accounted for by the Stark effect, while it could not be justified for other modulations that should affect all excitons in the same way.

Our data also give us the unique opportunity to analyze the dynamics of free charge-carriers in one dimension. Due to the linear dependence of the Δ*T*/*T* signal on pump fluence (inset Fig. 2c and Supplementary information), the most probable mechanisms of charge photogeneration remain either direct excitation or ultrafast linear exciton dissociation. According to our analysis, the charge-carrier population decay is monitored by the Δ*T*/*T* time trace at 363 nm, which represents the Stark amplitude evolution. Figure 3a shows that this decay is very accurately reproduced by a power law (Δ*T*/*T*)*_norm_* = *A* · *t*^−1/2^. The same power law is observed at different pump intensities (Supplementary material), thus excluding that the non-exponential kinetics is due to bimolecular non-geminate carrier annihilation. A monomolecular power law decay is the predicted dynamics for geminate recombination of free particles after random walk in an infinite one-dimensional chain^41^. In particular, the probability Ω(*t*) to survive at geminate recombination at delay *t* is expressed by 
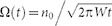
, where *W* is the diffusion rate and *n*_0_ = *L*_0_/*a*_0_ is the normalized initial particle separation, being *L*_0_ the particle distance and *a*_0_ the unit cell^41^. Considering that a hopping model is a good approximation for the high-mobility diffusive transport of SWNTs^42^,^43^, we estimate the diffusion rate W as the inverse of the scattering time *τ* = *m_av_μ*/(0.32*e*), where *m_av_* is the effective mass and *μ* is the charge-carrier mobility^44^. Using the value for mobility in a (6,5) SWNT of *μ* ~ 10^3^ *cm^2^*/*Vs* (diameter of ≈1 nm)^44^ and assuming *a*_0_ ~ 1 *nm*, we obtain *L*_0_ ~ 5 *nm*, meaning that the initial distance between a geminate electron-hole pair is of the same order of magnitude of the exciton correlation distance^8^. This suggests that “instantaneous” (<50 fs) linear exciton dissociation is the most likely mechanism of charge photogeneration. Possibly this process is favoured by the presence of atmospheric contamination, due to water and/or oxygen, which strongly reduces the exciton binding energy^45^,^46^,^47^,^48^,^49^.

In conclusion, we identified the transient energy shift of the S_33_ transition as a sensitive fingerprint of charge-carriers in (6,5) SWNTs. The assignment is based on the notion that photogenerated charge-carriers give rise to strong electric fields that in turn shift the energy of the more polarisable transition, namely the S_33_ resonance. Modulation spectroscopy through coherent phonons allows a precise measurement of the Stark shift of the S_33_ exciton, from which we discuss its binding energy in comparison with lower energy excitonic transitions. Our results indicate that charge-carriers are formed even upon pumping the first exciton, S_11_. This is a surprising outcome, since in principle S_11_ states are strongly bound and located well below the electron-hole continuum. The decay of the photogenerated charge-carrier population is well described by a model for geminate recombination in 1D. From the model, we estimate an initial charge-carrier separation of the same order of the exciton correlation length. This sheds additional light onto the generation mechanism, suggesting that the nascent excitons dissociate spontaneously, perhaps in presence of extrinsic screening of the Coulomb attraction due to water or other contamination. This result implies that charge photogeneration in SWNTs can be engineered, for instance for applications in optoelectronics, by manipulation of the tube environment. Long-lived charge carriers can only be obtained by promoting intertube separation, to escape efficient geminate recombination.

## Methods

The sample used for these investigations is highly enriched in the (6,5) species and embedded in a gelatin film. This film was prepared from 30 microliters of a density gradient ultracentrifugation (DGU) enriched SWNT suspension in a sodium cholate (SC)/sodium dodecyl sulfate (SDS) mixture^50^. Iodixanol as well as SDS residues from the DGU process were removed by dilution with SC solution and filtration with a benchtop centrifuge. The resulting suspension with 30 microliters volume was then mixed with 20 microliters of 15 wt% gelatin solution and finally drop-cast on a thin glass substrate. Ultrafast pump-probe spectroscopy was carried out on a very broad wavelength region from 340 nm to 1.1 μm, thus probing the transient absorption signal of the third (S_33_), second (S_22_) and first (S_11_) excitonic transitions of the sample (Fig. 1b). We excited the sample either with a broad IR pulse, peaked around 1 μm and with a transform-limited pulse duration of less than 15 fs (for measurements in Fig. 2 and 3) or with a 10-nm bandwidth pulse peaked at 570 nm (for measurements in Fig. 1b). As a probe we used: i) the second harmonic of a visible optical parametric amplifier (OPA), in order to achieve an overall temporal resolution of ≈50 fs in the probe region from 340 nm to 370 nm; ii) broadband white light super-continuum generated in CaF_2_ in the probe region from 340 nm to 650 nm and iii) broadband white light super-continuum generated in a sapphire plate in the probe region from 850 nm to 1.1 μm. We measured the differential transmission (Δ*T*/*T*) through the sample with an optical multichannel analyzer working at the full repetition rate (1 kHz) of the laser source^51^.

## Author Contributions

All authors discussed the results and implications and commented on the manuscript at all stages. G.S. and F.S. performed the pump-probe experiments, T.H. prepared the CNT sample, G.S. and D.V. performed the modelling of the pump-probe data. T.H. supervised the sample preparation, G.C. and G.L. supervised the pump-probe experiments and interpretation of the data.

## Supplementary Material

Supplementary Information Supplementary Information
