# Supplementary material for: High energetic excitons in carbon nanotubes directly probe charge-carriers
Source: Sci Rep. 2015 May 11;5:9681. doi: 10.1038/srep09681 (PMC4426596; doi:10.1038/srep09681)
Supplement: Supplementary Information [file srep09681-s1.pdf]

Supplementary material for

High energetic excitons in carbon nanotubes directly probe charge-carriers

Giancarlo Soavi[1], Francesco Scotognella,[1,2,3], Daniele Viola[1], Timo Hefner[4], Tobias Hertel[4], Giulio Cerullo [1, 2*] and Guglielmo Lanzani[1, 3†]

[1] *Dipartimento di Fisica, Politecnico di Milano, Piazza L. da Vinci 32, 20133 Milano, Italy*

[2] *IFN-CNR, Piazza L. da Vinci, 32, 20133 Milano, Italy*

[3] *Center for Nano Science and Technology@PoliMi, Istituto Italiano di Tecnologia, Via Giovanni Pascoli, 70/3, 20133 Milano, Italy*

[4] *Inst. for Physical and Theoretical Chemistry Dept. of Chemistry and Pharmacy, University of Wuerzburg, Wuerzburg 97074, Germany*

S1. Interpretation of the $\Delta T/T$ signal, temperature and pump photon energy dependence

Although it is commonly agreed that the first derivative of the ground state absorption spectrum can reproduce many of the features observed in the $\Delta T/T$ spectra of semiconducting SWNTs, the physical origin of this peculiar signal is still matter of debate. From a general point of view, a first derivative of the absorption spectrum can be ascribed to a shift of the excitonic transitions. This consideration can only exclude excited state absorption (ESA) as a possible origin of the observed signal, considering that many of the observed photoinduced absorption (PA) bands do not match with any possible transition from excited states [1]. The most evident demonstration of this assumption is indeed the $\Delta T/T$ signal of S_{33}, where a sharp PA band appears at approximately 360 nm (≈ 3.44 eV). If we consider that, after initial relaxation processes, all the photoexcited population is on S_{11}, thus at approximately 1 μm (≈ 1.24 eV), the observed ESA transition would end up in a state at approximately 265 nm (≈ 4.68 eV). Although we can definitely exclude ESA, the derivative shape in the $\Delta T/T$ signal can arise from many different photoexcited species, ranging from bi-excitons [2] to trions [3], thermal effects [4] and Stark effect [5].

To exclude thermal effects we performed temperature dependent measurements using a cryostat equipped with a liquid nitrogen reservoir. We observed that the signal is weakly sensitive to changes in temperature (Fig. S1). A very small red-shift (approximately 2 nm when moving from 300 K to 130 K) of the $\Delta T/T$ zero (and thus of the ground-state absorption spectrum) can be detected for decreasing temperatures. This excludes geometrical re-arrangement (i.e. diameter distortion) and thermal effects as a possible origin of the observed signal, as the increase in temperature is extremely weak in TA experiments at 1 kHz [5] and the $\Delta T/T$ signal is reproduced by a shift of more than 10 nm. To exclude bi-excitons and trions we performed measurements with different pump-photon energies on a very broad probe region, ranging from 340 nm to 650 nm. We notice that: i) we always obtain the same derivative shape both for resonant and non-resonant excitation with respect to the excitonic transitions (Fig. S2); ii) we observe a clear derivative shape in a probe region far from any excitonic peak (Fig. S2). The first observation (i) rules out bi-excitons as a possible explanation, as we expect that more excitons, and consequently bi-excitons, are formed with resonant excitation. Trions, instead, can arise when a charge is photogenerated from the pump pulse and thus can also appear with non-resonant excitation. The second observation (ii) points out that the whole ground state absorption spectrum (and not only excitons) undergoes a red shift upon photoexcitation. This excludes both bi-excitons and trions, whose appearance should depend selectively on the excitonic transitions. On the contrary, the Stark effect lacks this selectivity and simply modifies the whole absorption spectrum.

The interpretation of the derivative shape in the $\Delta T/T$ as induced by Stark effect can be successfully extended to other modulation spectroscopy experiments. For example, from the analysis of the signal on S_{33} it appears that the presence of a dense continuum of states plays a fundamental role in the $\Delta T/T$ spectra and thus we expect to find a similar derivative shape already on S_{11} for those tubes with small binding energy, such as metallic tubes. This experiment was performed on isolated metallic CNTs by Gao *et al.* [6], resulting in the expected derivative shape which was, instead, interpreted in terms of bi-excitons. Similarly, Ham *et al.* [7] found a surprisingly high electro-absorption signal in the UV region with respect to the first and second excitonic subbands, which was further enhanced by the presence of metallic CNTs. This discussion deserves at least two additional comments. First, the red-shift of the ground state absorption spectrum induced by bi-excitons or trions is determined by their binding energy and thus we would expect a bigger red-shift for strongly bound excitons [8] such as S_{11} with respect to S_{33} or, similarly, for semiconducting SWNTs with respect to metallic ones. On the contrary, the red-shift due to Stark effect is inversely proportional to the exciton binding energy [9, 10], in accordance with the experimental evidences. These considerations further exclude bi-excitons and trions as a possible

explanation for the observed $\Delta T/T$ signal. Second, the nature of S_{33} in semiconducting SWNTs is still matter of debate. Rayleigh scattering experiments have demonstrated that it is consistent with an excitonic model [11], nevertheless it shows features ascribable to unbound electron-hole pairs [12, 13], in contrast with theoretical studies which predict high binding energies [14]. Our results confirm that S_{33} has a low binding energy, in analogy with M_{11} of metallic tubes.

Finally, we check that the power law decay dynamics of the 363 nm signal is independent from the excitation fluence, thus excluding bimolecular recombination processes (Fig. S3).

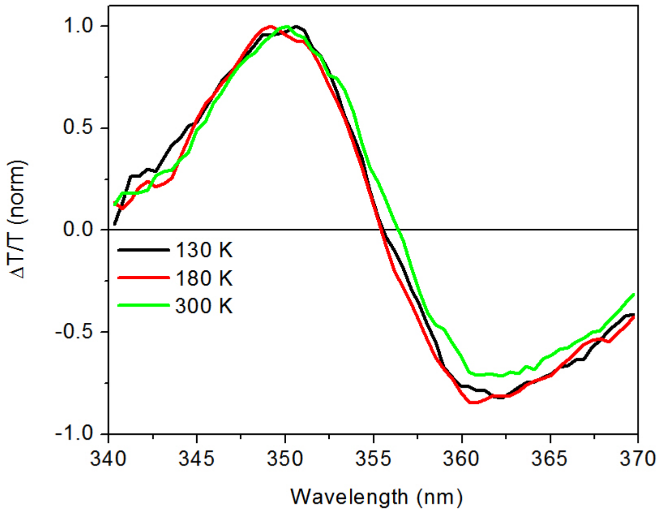

Supplementary Figure S1. $\Delta T/T$ spectra at a pump-probe delay of 10 ps as a function of temperature. The sample was excited with a broadband IR pulse while the probe was obtained by white light generation on a CaF_2 plate.

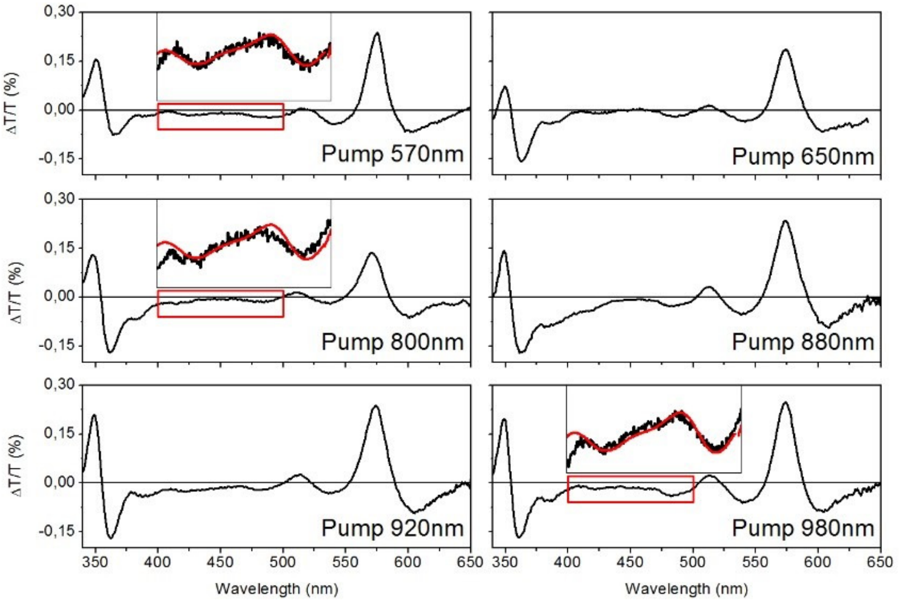

Supplementary Figure S2. $\Delta T/T$ spectra at a pump-probe delay of 30 ps for six different pump-photon energies, resonant and non-resonant with respect to the excitonic transitions of the (6,5) CNT sample under investigation. Here each pump pulse had 10 nm bandwidth while the probe was obtained by white light generation on a CaF_2 plate. For pump wavelengths of 570 nm, 800 nm and 980 nm we zoom the probe region between S_{22} and S_{33} (from 400 nm to 500 nm) and we fit it with the $\Delta T/T$ obtained from a 100 meV shift of the ground state absorption spectrum [5].

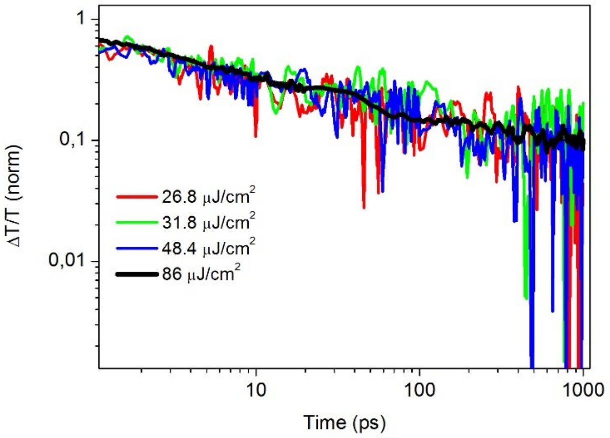

Supplementary Figure S3. Dynamics at 363 nm probe wavelength and broadband IR excitation for different pump fluences.

S2. Fitting procedure

In Fig. S4 we compare experimental results with our fitting model. For any pump-probe delay we calculate the ΔA transient spectra from $\Delta T/T$, with $A = -\ln(T)$, as a function of the probe energy (instead of wavelength). This procedure is useful in order to have a direct comparison with the O.D. of the ground state absorption spectrum. Each spectrum (namely each ΔA at a fixed pump-probe delay) is reproduced by the sum of three contributions: $\Delta A_{fit} = \Delta A_{bleach} + \Delta A_{Stark} + K$. First, we run the fitting routine for each ΔA spectrum (i.e. at different pump-probe delays) and we set the shape (i.e. peak position and broadening) of ΔA_{bleach} and ΔA_{Stark} from the average of the values obtained for each pump-probe delay. After this, the shape is kept constant and we only vary its amplitude as a function of the pump-probe delay. We thus obtain both spectral information and dynamics (the evolution of the amplitude of each component) of the three signals. We decided to keep the shape of ΔA_{bleach} and ΔA_{Stark} fixed since, from the first fitting routine, the parameters undergo only slight changes without any specific temporal trend. In particular: i) ΔA_{bleach} is obtained from a Lorentzian function, typical of excitonic transitions [11], peaked at 3.55 eV with 170 meV FWHM; ii) ΔA_{stark} is the difference between two Lorentzian functions with 170 meV FWHM peaked at 3.42 eV and 3.55 eV; iii) K is a constant over the entire probe energy region. Interestingly the constant K, which we attributed to excited state absorption to the continuum of states [5], shows exactly the same dynamics of the PB signal, indicating that it arises from S_{11}. This PA band was already observed at low energies (≈ 300-400 meV), setting a lower limit for the exciton binding energy [15].

Finally, we convert again our model to $\Delta T/T$ as a function of the probe wavelength. The large broadening of the Lorentzian excitonic function for high energetic excitons is in good agreement with recent experiments [16] and might be additionally altered by environmental effects [17]. This kind of analysis is necessary in order to separate the different contributions arising from excitons (PB and PA) and charge-carriers (Stark effect). From the study of the obtained dynamics (Fig. S4), we observe that the three components are formed within our temporal resolution (≈ 50 fs). The linear trend of the signal with respect to the pump fluence excludes non-linear processes such as two-photon absorption or exciton-exciton annihilation [3]. Thus, the most feasible mechanisms for charge-carrier photogeneration remain direct excitation or ultrafast linear exciton dissociation. Direct excitation is easily accessible with high energetic photons [9] while possible mechanisms for exciton dissociation are still largely discussed. Recent experiments [18] show that S_{22} will more likely undergo dissociation into free electron-hole pairs

with respect to relaxation into S_{11}. Nevertheless, S_{22} lies in the continuum of states and thus exciton dissociation processes are more likely, while dissociation of S_{11} was predicted to occur only in presence of intense external electric fields [19]. Experiments in this direction are indeed controversial, showing both field-induced exciton dissociation [20, 21] or linear exciton dissociation and instantaneous free carrier generation [5, 22, 23].

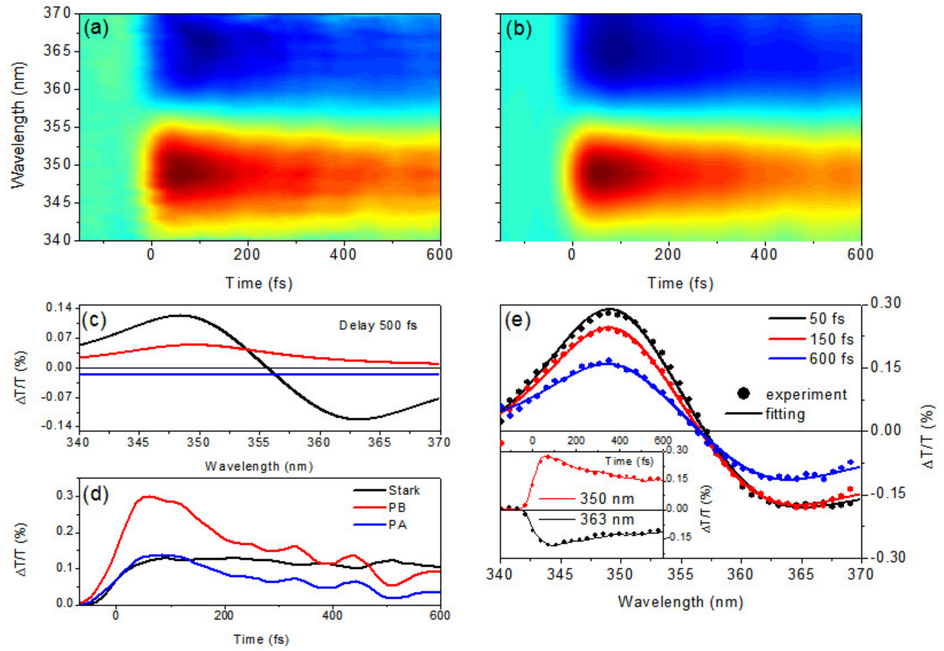

Supplementary Figure S4. Comparison between experimental (a) and fitted (b) $\Delta T/T$ map. (c) Spectra and their amplitude/ dynamics (d) (inset) used for the fit. (e) Comparison between the fit and experimental data.

S3. Bi-excitons and Stark effect

Both bi-exciton and Stark shift are Coulomb related phenomena, and bear similarity. For this reason it is worth to further discuss the experimental data in order to confirm that the observed red-shift of the absorption spectrum actually arises from Stark effect.

1. **Different dynamics between S_{11} or S_{22} and S_{33}.** The interpretation of the pump-probe signal as a bi-exciton implies that that the decay dynamics are the same for the S_{11}, S_{22} and S_{33} transitions, given that the excitation conditions are the same. In particular, they will follow the decay dynamics of excitons since the pump pulse will only generate excitons and their lifetime will determine the

probability for the probe pulse to generate a bi-exciton. This is not what we observe in our experiments. When we compare the decay dynamics at the peak of the bleaching for S_{22} (570 nm) and S_{33} (350 nm) under *exactly the same experimental conditions* (broadband IR pump at resonance with S_{11} and simultaneous probe of S_{22} and S_{33} with white light generated in CaF_2), we find a faster decay for S_{22} compared to S_{33} (Fig. S5). A power law decay dynamics was observed also for the S_{11} excitonic transition in ensembles of semiconducting SWNT [24, 25] after approximately 10 ps. This fully supports our interpretation since S_{11}, according to our model, will decay as others ($t^{-0.5}$), once excitonic features are decayed. Moreover, in Ref. 24 and 25 due to spectral congestion, detailed assignment of the dynamics is very difficult and spectral overlap may affect the kinetics. The observed dynamics were assigned to either bimolecular triplet [24] or singlet [25] annihilation. These assignments are in contrast with our work, which shows for the first time a diffusion limited geminate recombination in a single chirality specimen and in the linear excitation regime. Our analysis of the spectral shape of the S_{33} $\Delta T/T$ signal allows to assign the transient signal to charge carrier recombination, enlightening previous results. Charge carriers are never mentioned in Ref. 24 and 25 but our work clarifies that charge carriers were indeed involved in those cases as well.

2. **Derivative shape is independent from excitation wavelength**. we have shown that the derivative shape over a very broad probe wavelength region is independent from the pump wavelength (also in agreement with Ref. 22). The observed energy shift depends on the local field that in turns depends on the local geometry, but enhancing the number of charges leads to more modulation sites, not to a larger modulation. In the experiments, the fluence for each excitation wavelength was adjusted in order to obtain the same transient absorption signal. Nevertheless, since we study the shape of the $\Delta T/T$ signal (the energy-shift) and not its amplitude, the number of photogenerated charges is not of primary importance. To explain this in term of bi-excitons, instead, we need to assume that any excitation wavelength leads to the formation of S_{11} excitons but instead we know that: i) S_{11} excitation generates also charges [5, 22]; ii) S_{22} excitation generates charges with high yield due to spontaneous exciton dissociation [18]; iii) above-gap out of resonance excitation does not give rise to excitons, but on the other hand it has been shown to generate charges.
3. **Red-shift affects also a spectral region far from excitons**. In our previous work [5] we showed that the red-shift of the absorption spectrum affects also a spectral region far from excitonic transitions. This can be observed also in Figure S2, where we zoom in on the region between 400 nm and 500 nm and we show (red line) the fit obtained by a simple red-shift of the absorption

spectrum. In this probe region there are no excitonic transitions related to the (6,5) SWNTs and thus the signal cannot be attributed to bi-excitons. These spectral features can be easily interpreted in terms of charge-induced Stark effect since the induced electric field lacks selectivity and acts on the whole absorption spectrum.

4. **Binding energy of the biexciton is not consistent**. In the case of bi-exciton the red-shift of the absorption spectrum can be used to derive its binding energy [26] and we expect that a bi-exciton formed by S_{11}+S_{22} ("excited bi-exciton") is more stable than a S_{11}+S_{33} bi-exciton. This would result in larger red-shifts on S_{22} with respect to S_{33}, in contrast with our experimental evidences that show exactly the opposite trend. In fact the shift on S_{22} is always smaller than the value obtained for S_{33}. Considering the calculation by Pedersen *et al.* [27] for the S_{11}-S_{11} bi-exciton we can estimate that $E_{12} > E_{13}$ (where $E_{xy} = E_x + E_y - E$ is the binding energy of the excited bi-exciton, E_x and E_y are the binding energies of the single excitons and E is a positive constant) if we assume that $E_2 > E_3$ [12]. On the other hand, the Stark effect is consistent with a larger red-shift on S_{33} with respect to S_{22} since it scales like the inverse of the exciton binding energy.

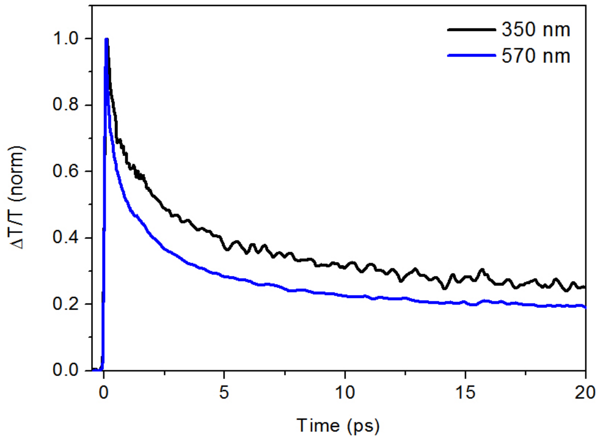

Supplementary Figure S5. Normalized decay dynamics of the S_{33} (350 nm, black line) and S_{22} (570 nm, blue line) excitons for broadband IR excitation resonant with S_{11}.

S4. Charge induced screening and Stark effect

Photogenerated charge-carriers may also lead to enhanced screening. Moreover, both carrier-induced screening and Stark effect have the same effect (red- or blue-shift) on the optical transitions. However, Stark effect is more suitable to explain our results, as we discuss in the following. First, as we have already noticed, the red-shift affects also the energy region between S_{22} and S_{33}, thus far from

excitonic optical transitions; this shift cannot be explained by carrier induced screening. Second, we can compare our results with recent studies that involve carrier induced screening of excitons:

1. Steiner *et al.* [28] observe, for a relatively small shift (20 meV) of the excitonic transition, a drastic reduction of the optical absorption. This observation is not consistent with our experimental data, since the simultaneous presence of a red-shift and a reduction of the absorption would result in a *strongly asymmetric* "derivative-like" shape of the transient absorption spectra (in other words large bleaching + small red-shift), which is clearly not the case in our experiments (the signal is dominated by red-shift and the shape is almost perfectly symmetric).
2. Spataru *et al.* [29] predict very small changes in the optical transitions (they predict a blue-shift of S_{11} and S_{22}) upon large doping, while the main effect in their theoretical calculations regards a band-gap renormalization of the electronic band-structure. Moreover, according to their calculations the effect of doping is stronger on S_{11} with respect to S_{22}. This last point, in particular, is in strong contrast with our experimental results, that show a stronger shift (always a red-shift) for higher energetic excitons.

Instead, Stark effect can explain all our experimental observations: (i) the local electric field acts on the whole absorption spectrum, including the energy region far from excitons; (ii) the large red-shift arises from the extremely intense local electric field due to photogenerated charge-carriers; (iii) the red-shift is stronger for high energetic excitons since they have smaller binding energy (as we discuss in the manuscript).

<u>S5. Pump fluence and linearity</u>

The evidence of a charge photogeneration yield that is linear with respect to the pump fluence for S_{11} excitation is in agreement with optical pump – THz probe experiments [22, 23]. Similarly, Kumamoto *et al.* [18] found that S_{22} spontaneously dissociates into charge-carriers. Of course, also non-linear processes such as two-photon absorption or exciton-exciton annihilation might occur at higher excitation fluences and be responsible for charge photogeneration, but the two different excitation scenarios do not exclude each other. Exciton-exciton annihilation is expected to occur in the saturation regime, thus with a sub-linear dependence with respect to the pump fluence. In particular, experiments by Yuma *et al.* [30] pointed to the description of charges (through the detection of trions) on S_{11}, which retains a strong excitonic behaviour and it is thus not the ideal probe for charge-carriers. It might be that when probing the

S_{11} exciton a higher charge photogeneration yield, enhanced by the presence of non-linear mechanisms, improves the detectability of charge-related phenomena. This, instead, is not necessary on S_{33}, which turns out to be the ideal probe for charge-carriers. In our experiments the shape of the ΔT/T signal (fig. 2c in the main text) is the same for all the different fluences, thus confirming that even at lower fluences (and thus in the linear regime) the charge induced Stark effect dominates the S_{33} signal. If charge photogeneration would be limited to the saturation regime (thus arising from exciton-exciton annihilation), we would instead expect the appearance of the derivative shape only at higher fluences, which is obviously not the case.

Moreover, we point out that the saturation of the ΔT/T signal might arise also from saturation of the pump-pulse absorption. This is also the case of ref. [30], as they observe at page 5: *"The saturation mechanism observed here cannot be totally explained by the EEA and suggests a saturation on the pump pulse absorption"*.

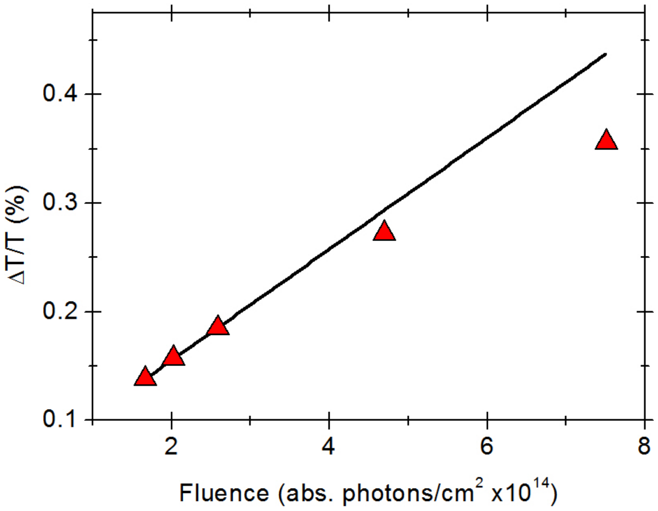

Supplementary Figure S6. Absolute value of the $\Delta T/T$ peak signal at 363 nm as a function of the pump fluence (absorbed photons/cm^2). The linear fit is performed on the first three points.

S6. Fourier transform of the RBMs

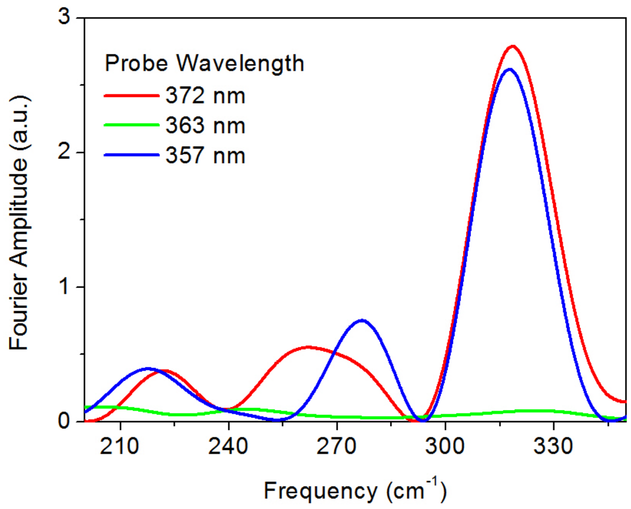

Supplementary Figure S7. Fourier Transform of the oscillating component of the ΔT/T signal for three different probe wavelengths: the two maxima (372 nm and 357 nm) and the zero (363 nm) amplitude of figure 3a of the main text. The peak of the FT is at 318 cm^{-1}.

S7. Determination of the initial electron-hole distance L_0

In order to calculate the initial electron-hole distance L_0 we proceed as follows. We fit the normalized differential transmission with a power law decay $\frac{\Delta T}{T} = A \cdot t^{-0.5}$, which can be interpreted as the probability to survive at geminate recombination for a delay t. This is given, from ref. [31], by $\Omega(t) = n_0/\sqrt{2\pi W t}$. With simple algebra we have $n_0 = A \cdot \sqrt{2\pi W}$, being W the diffusion rate, $n_0 = L_0/a_0$ the normalized initial particle separation (L_0 is the initial particle distance and a_0 a characteristic unit of length) and $A \sim 0.8 \cdot 10^{-6} s^{1/2}$ a fitting parameter. We estimate the diffusion rate W as the inverse of the scattering time $\tau = m_{av}\mu/(0.32e)$, where m_{av} is the effective mass and μ is the charge-carrier mobility [32], in the order of $\mu \sim 10^3 - 10^4\ cm^2/Vs$ for SWNTs [32]. With these values we obtain $n_0 \sim 1.5 - 5$, and thus $L_0 \sim 1.5 \cdot a_0 - 5 \cdot a_0$. We now need to estimate the characteristic length a_0. Our model describes a random walk recombination on a one-dimensional space by introducing a hopping rate. This is associated to a jump length that is parametrized on the metrics of the space. From the physical point of view, this implies a lattice of sites that the particle occupy during propagation. Often the space geometry is not periodic, due to disorder. In a disordered crystals of well-defined physical units, like a molecular solid, the average inter unit (intermolecular) distance is an obvious choice for the jump length. In a crystal where polaronic states are propagating, the correct metrics to be adopted is not trivial, and it is associated

to the polaron size. In the case of SWNTs, we think the correct order of magnitude is the fundamental cut-off length of the 1D space, of the order of the diameter of the tube, thus $a_0 \sim 1\ nm$. Alternatively, the exciton correlation size, as previously measured, could suggest the jump length as 2 nm. In both cases we find that the initial distance is not very large, far smaller than the tube size, and consistent with the idea of an excitonic transition to an initial state that is ionized and undergoes geminate recombination, according to the kinetic found.

Supplementary References

[1] B. Gao, G. V. Hartland and L. Huang, ACS nano **6**, 5083 (2012).

[2] D. J. Styers-Barnett, S. P. Ellison, B. P. Mehl, B. C. Westlake, R. L. House, C. Park, K. E. Wise and J. M. Papanikolas J. Phys. Chem. C **112**, 4507 (2008).

[3] S. M. Santos, B. Yuma, S. Berciaud, J. Shaver, M. Gallart, P. Gilliot, L. Cognet and B. Lounis Phys. Rev. Lett. **107**, 187401 (2011).

[4] T. Koyama, S. Yoshimitsu, Y. Miyata, H. Shinohara, H. Kishida and A. Nakamura, J. Phys. Chem. C **117**, 20289 (2013).

[5] G. Soavi, F. Scotognella, D. Brida, T. Hefner, F. Späth, M. R. Antognazza, T. Hertel, G. Lanzani and G. Cerullo, J. Phys. Chem. C **117**, 10849 (2013).

[6] B. Gao, G. V. Hartland and L. Huang, J. Phys. Chem. Lett. **4**, 3050 (2013).

[7] M.-H. Ham, B.-S. Kong, W.-J. Kim, H.-T. Jung and M. S. Strano, Phys. Rev. Lett. **102**, 047402 (2009).

[8] D. Kammerlander, D. Prezzi, G. Goldoni, E. Molinari and U. Hohenester, Phys. Rev. Lett. **99**, 126806 (2007).

[9] J. Crochet, S. Hoseinkhani, L. Lüer, T. Hertel, S. K. Doorn and G. Lanzani, Phys. Rev. Lett. **107**, 257402 (2011).

[10] M. S. Arnold, J. L. Blackburn, J. Crochet, S. K. Doorn, J. G. Duque, A. Mohitee and H. Telg, Phys. Chem. Chem. Phys. **15**, 14896 (2013).

[11] S. Berciaud, C. Voisin, H. Yan, B. Chandra, R. Caldwell, Y. Shan, L. E. Brus, J. Hone and T. F. Heinz, Phys. Rev. B **81**, 041414 (2010).

[12] P. T. Araujo, S. K. Doorn, S. Kilina, S. Tretiak, E. Einarsson, S. Maruyama, H. Chacham, M. A. Pimenta and A. Jorio, Phys. Rev. Lett. **98**, 067401 (2007).

[13] T. Michel, M. Paillet, J. C. Meyer, N. V. Popov, L. Henrard and J.-L. Sauvajol, Phys. Rev. B **75**, 155432 (2007).

[14] C. D. Spataru, S. Ismail-Beigi, L. X. Benedict and S. G. Louie, Phys. Rev. Lett. **92**, 077402 (2004).

[15] Y.-Z. Ma, L. Valkunas, S. M. Bachilo and G. R. Fleming, J. Phys. Chem. B **109**, 15671 (2005).

[16] E. H. Haroz, S. M. Bachilo, R. B. Weisman and S. K. Doorn, Phys. Rev. B **77**, 125405 (2008).

[17] J.-C. Blancon, M. Paillet, H. N. Tran, X. T. Than, S. Aberra Guebrou, A. Ayari, A. San-Miguel, N.-M. Phan, A.-A. Zahab, J.-L. Sauvajol *et al.*, Nat. Commun. **4**, 2542 (2013).

[18] Y. Kumamoto, M. Yoshida, A. Ishii, A. Yokoyama, T. Shimada and Y. K. Kato, Phys. Rev. Lett. **112**, 117401 (2013).

[19] V. Perebeinos and P. Avouris, Nano Lett. **7**, 609 (2007).

[20] A. D. Mohite, P. Gopinath, H. M. Shah and B. W. Alphenaar, Nano Lett. **8**, 142 (2008).

[21] A. Mohite, J.-T. Lin, G. Sumanasekera and B. W. Alphenaar, Nano Lett. **6**, 1369 (2006).

[22] M. C. Beard, J. L. Blackburn and M. J. Heben, Nano Lett. **8**, 4238 (2008).

[23] S. A. Jensen, R. Ulbricht, A. Narita, X. Feng, K. Müllen, T. Hertel, D. Turchinovich and M.Bonn, Nano Lett. **13**, 5925 (2013).

[24] R. Russo, E. Mele, C. Kane, I. Rubtsov, M. Therien and D. Luzzi, Phys. Rev. B **74**, 041405(R) (2006).

[25] J. Allam, M. T. Sajjad, R. Sutton, K. Litvinenko, Z. Wang, S. Siddique, H. Q. Yang, W. H. Loh and T. Brown, Phys. Rev. Lett **111**, 197401 (2013).

[26] V. I. Klimov, Annu. Rev. Phys. Chem. **58**, 635 (2007).

[27] T. G. Pedersen, K. Pedersen, H. D. Cornean and P. Duclos, Nano Lett. **5**, 291 (2005).

[28] M.Steiner, M. Freitag, V. Perebeinos, A. Naumov, J. P. Small, A. A. Bol, and P. Avouris, Nano Lett. **9**, 3477 (2009)

[29] C. D. Spataru and F. Léonard, Phys. Rev. Lett. **104**, 177402 (2010).

[30] B. Yuma *et al.*, Phys. Rev. B **87** 205412 (2013).

[31] I. V. Zozulenko, Solid State Commun. **76**, 1035-1040 (1990).

[32] X. Zhou, J.-Y. Park, S. Huang, J. Liu, P. McEuen, Phys. Rev. Lett. **95**, 146805 (2005).
